# Atomistic simulations of the optical absorption of type-II CdSe/ZnTe superlattices

**DOI:** 10.1186/1556-276X-7-543

**Published:** 2012-10-02

**Authors:** Soline Boyer-Richard, Cédric Robert, Lionel Gérard, Jan-Peter Richters, Régis André, Joël Bleuse, Henri Mariette, Jacky Even, Jean-Marc Jancu

**Affiliations:** 1 Université Européenne de Bretagne, INSA, FOTON, UMR 6082, Rennes, 35708, France; 2 Nanophysics and Semiconductors Group, Institut Néel-CNRS, Grenoble, 38042, France; 3 Nanophysics and Semiconductor Group, CEA/Université Joseph Fourier, CEA/INAC/SP2M, Grenoble, 38054, France

**Keywords:** Type-II transition, Superlattice, ZnTe/CdSe, Absorption, Tight-binding, 73.21.Cd, 78.67.Pt, 78.66.Hf

## Abstract

We perform accurate tight binding simulations to design type-II short-period CdSe/ZnTe superlattices suited for photovoltaic applications. Absorption calculations demonstrate a very good agreement with optical results with threshold strongly depending on the chemical species near interfaces.

## Background

**Figure 1:**
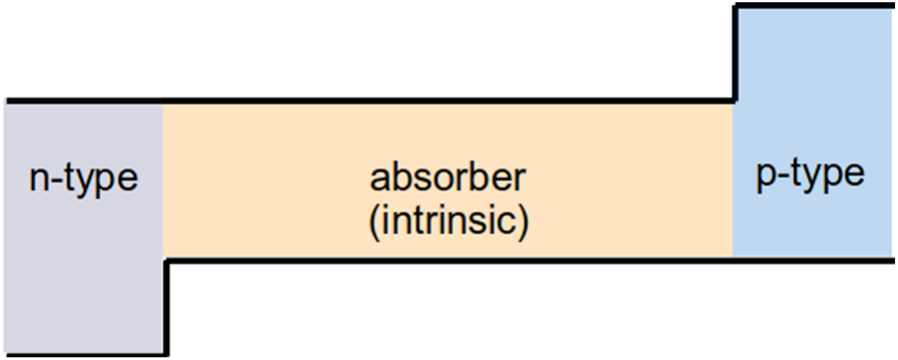
Schematic band alignment of a p-i-n structure for photovoltaic application.

A photovoltaic cell is typically built onto three parts: a light absorber surrounded by an n-type and a p-type layer to separate and collect the photo-generated charge carriers (Figure 1). In an ideal case, the n-type layer conduction band shall be aligned with the conduction band of the absorber while forming a barrier for holes in the valence band. Respectively, the p-type layer valence band shall be aligned with the valence band of the absorber while forming a barrier for electrons in the conduction band. Such a three-material system does not exist for semiconductors, and we propose here to mimic it by using a type-II short-period superlattice (SL) made of two materials with a type-II band alignment. The material with the lowest conduction band will then be used as the n-doped contact and the other one as the p-doped contact. Type-II material systems built with III-V semiconductors are already available and mainly known for mid-infrared detectors (for a review see [1]). CdSe and ZnTe bulks have been chosen to this scope because they are almost lattice-matched and exhibit a type-II interface. Furthermore, this SL first optical transition value can be designed to emphasize the solar spectrum absorption. In this letter, we propose atomistic modeling of the optical absorption of type-II CdSe/ZnTe superlattices. The ZnTe and CdSe layer thicknesses are optimized to maximize absorption in the solar spectrum and threshold is studied as function of the interface-related properties. 

## Methods

### Tight-binding simulation of bulk materials

We consider the extended-basis sp3d5s* tight-binding (TB) model which has proved to provide a band structure description with a sub-milli-electron volt precision throughout the Brillouin zone of binary III-V semiconductors [2] including quantum heterostructures [3] and surfaces [4]. We model CdSe, CdTe, ZnSe, and ZnTe in a cubic phase by fitting both the experimental band parameters and the first-principle electronic structures in the GW approximation. Strain effects are taken into account in the same way of smaller TB models using a recent generalization of Harrison’s d2 law for hopping integrals known to be reliable for strained III-V quantum well structures [5]. The valence band offset (VBO) between the material constituents are taken from our own experimental measurements for the CdSe/ZnTe interface [6] and from *ab initio* modeling for the interfacial bonds [7]. Finally, the optical dipole matrix elements are derived from the TB Hamiltonian [8].

### Superlattice absorption calculation

We have performed TB calculations to design the most suited type-II CdTe/ZnSe [001] configuration which fully maximizes the absorption in the solar spectrum. Associated SL band structures are found very sensitive to the VBO between CdTe and ZnSe. As knowledge of this VBO is scanty, we have performed photoluminescence measurements on a simple ZnTe/CdSe interface as a function of incident power. The extracted value is of 0.74 ± 0.02 eV, which is slightly different from the experimental result of 0.64 eV [9], but in agreement with the *ab initio* calculations [7]. We have used a mesh of 1,200 points to sample the reduced Brillouin zone near the Γ-point. The discrete transitions are dressed with a Gaussian broadening of 0.005 eV to get smooth spectral functions. As CdTe and ZnSe do not share any common atom, three configurations have been simulated: CdTe-like or ZnSe-like terminations (symmetric D_2d_ SL) and the CdSe/ZnTe interfaces (non-symmetric C_2v_ SL).

## Results and discussion

We first test our TB model by calculating the electronic properties of non-symmetric (CdSe)_7_/(ZnTe)_7_ superlattices and found a strong in-plane anisotropy of the optical spectrum. The energy subbands are calculated at the Γ-point and labeled according to their dominant bulk-state component: conduction (e), heavy-hole (hh), and light-hole (lh).

**Figure 2:**
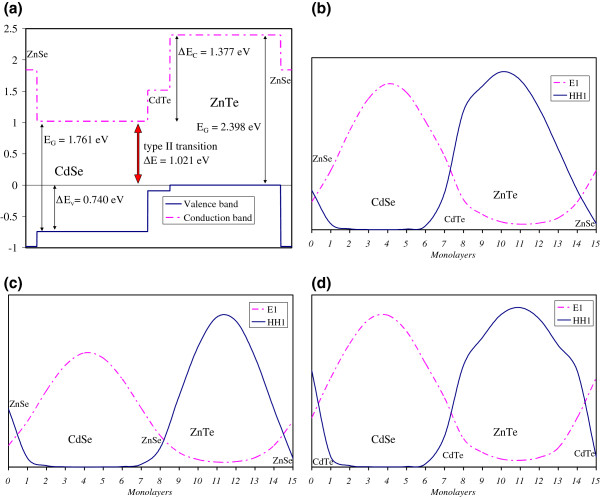
**Schematic diagram of the band alignment for the (CdSe)**_**7**_**/(ZnTe)**_**7**_**SL and electronic wave functions.** Schematic diagram of the band alignment for the (CdSe)_7_/(ZnTe)_7_ SL (**a**) and electronic wave functions of the upper valence miniband and the lower conduction miniband in three interfaces cases: (**b**) non-symmetric case, (**c**) ZnSe interfaces, and (**d**) CdTe interfaces. Envelope functions are plotted along the [001] axis (molecular average between the charge densities on cation and anion sites) for clarity reasons and to better evidence the location of electronic states in the structure.

Table 1 reports on the dipole matrix elements squared (*E*_P_ in electron volt) between the first Γ-like valence and conduction band states for transverse electric polarization in the CdSe/ZnTe superlattices. In a non-symmetric C_2v_ configuration, interfaces are characterized by forward and backward bonds lying in the (110) (or *x-*) and (−110) (or *y-*)planes respectively, giving the definition of optical axes here considered: [110] (*x*), [−110] (*y*), and [001] (*z*). In addition the growth sequence in the simulation is as follows: Se-Cd=Se-Cd=Se….Cd=Te-Zn=Te…where ‘-’ and ‘=’ indicate chemical bonds in the *x-* and *y-*planes, respectively. For the associated superlattice, we found for the fundamental transition a polarization degree $\left(\frac{E_{\mathrm{Py}}-E_{\mathrm{Px}}}{E_{\mathrm{Py}}+E_{\mathrm{Px}}}\right)$ of 17% (canceled for symmetric SL in agreement with point group D_2d_), and this is consistent with photoluminescence measurements [10]. As seen in Table 1, the e1-hh1 transition strongly depends on the chemical species at SL terminations, which underlines that relevant active states are mainly located in the surrounding of interfaces. Very interestingly, the CdTe-like terminations allow for a lower absorption threshold due to the very small VBO between CdTe and ZnTe. This explanation can be illustrated from the calculation of the charge densities as shown in Figure 2. Obviously, the ground-state wave function is maximized in CdTe layers compared to ZnSe. The CdTe termination mimics larger ZnTe layers increasing the energy level of hh1. This type of interface allows for a stronger overlap between the valence and conduction subbands, which enhances the optical matrix elements of the band edge. 

**Table 1:** Valence and conduction energy levels at Γ-point and dipole matrix element in transverse electromagnetic polarization

	**LH**_**1**_**(eV)**	**HH**_**1**_**(eV)**	**E**_**1**_**(eV)**	**Δ*E*(eV)**	***E***_**P*x***_**(eV)**	**Polarization**
Non-symmetric SL	−0.212	−0.062	1.340	1.402	3.08	17.5%
ZnSe termination	−0.255	−0.101	1.330	1.430	2.56	-
CdTe termination	−0.195	−0.048	1.292	1.339	3.24	-

The zero level is taken at the bulk CdSe valence band.

**Figure 3:**
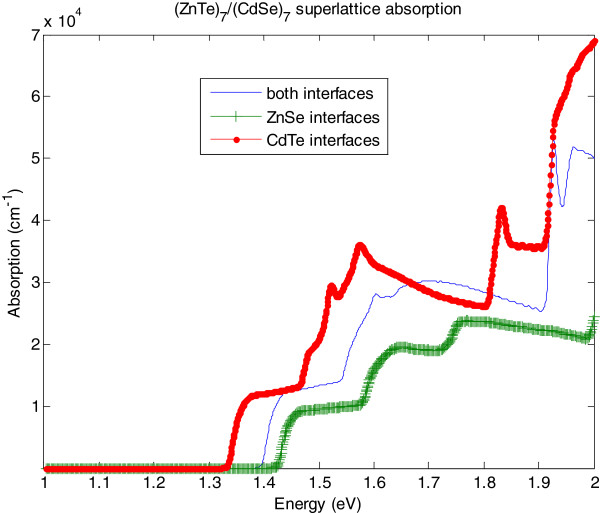
**Absorption coefficient of the (CdSe)**_**7**_**/(ZnTe)**_**7**_**SL for three types of interface, as a function of energy.**

**Figure 4:**
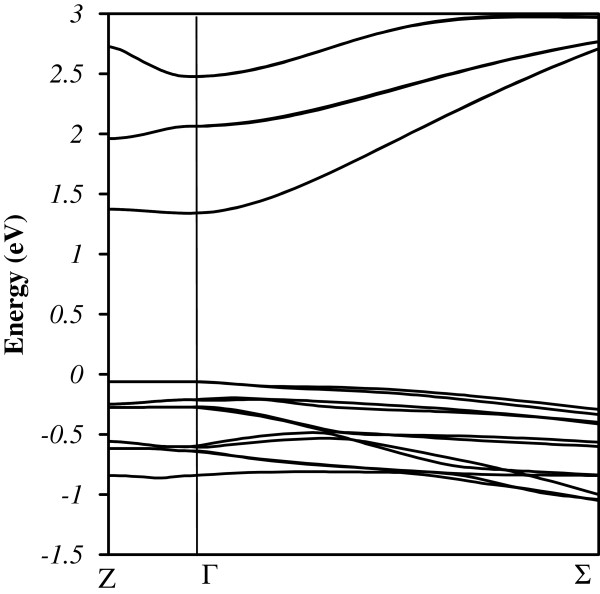
**Band diagram of the CdSe/ZnTe SL with non-symmetric interfaces.** Band diagram (black lines) of the CdSe/ZnTe SL with non-symmetric interfaces in the reduced Brillouin zone along the [001] and [110] directions.

**Figure 5:**
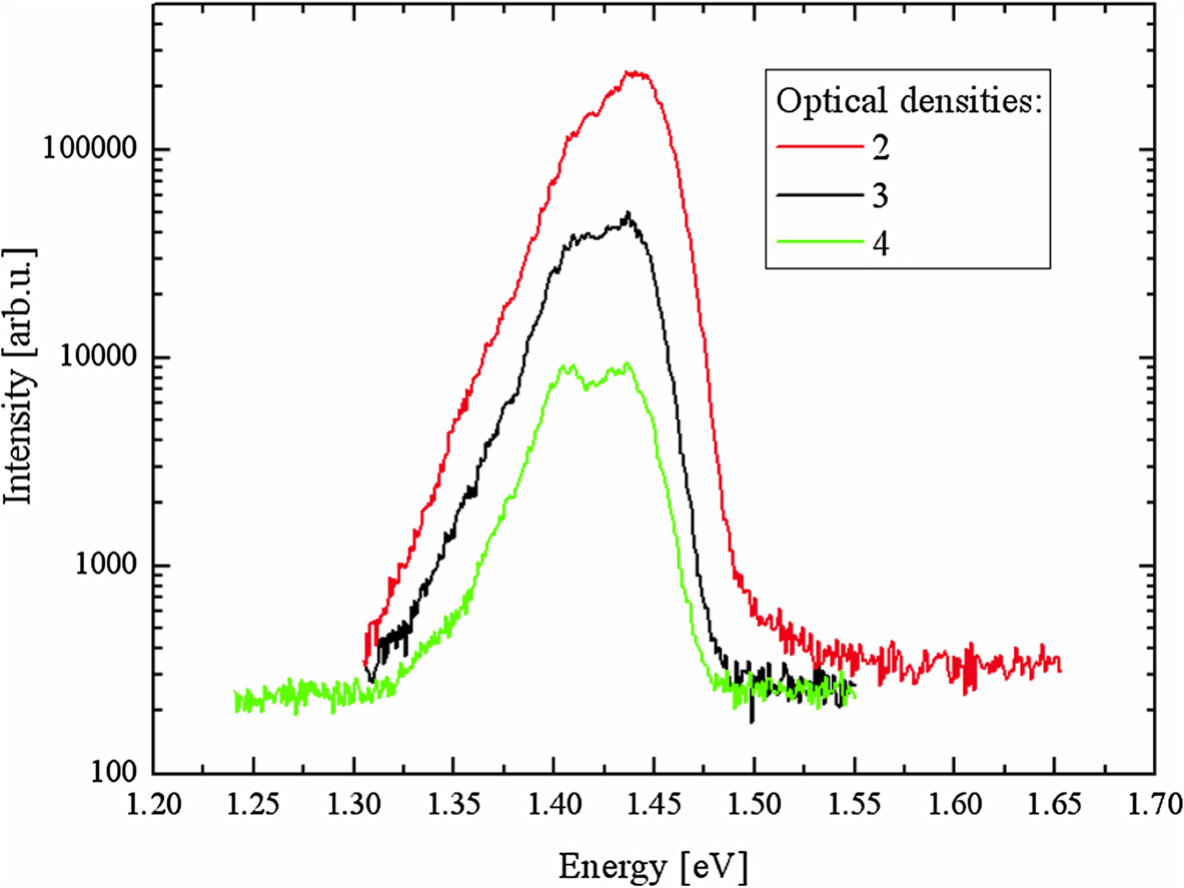
**Photoluminescence spectra of (CdSe)**_**7**_**/(ZnTe)**_**7**_**SL grown by MBE.**

Figure 3 shows the absorption coefficient calculated for each type of superlattice. In the simulation, we considered six conduction and 12 valence subbands. Consequently, the calculated spectral function is valid near the center of the reduced Brillouin zone up to 2 eV above the valence band maximum. In the same way of optical transitions, the absorption threshold is found strongly dependent on the chemistry at interfaces. According to these calculations, the CdTe interfaces should be favored to increase absorption in the solar spectrum. However, they are very difficult to control during the sample growth by molecular beam epitaxy (MBE). The major steps correspond to the different conduction minibands. The peaks around 1.52 and 1.83 eV for CdTe terminations, and around 1.9 eV for the non-symmetric SL, correspond to the curvature inversion observed in the valence miniband around −0.6 eV for the non-symmetric SL as shown in Figure 4. Absorption measurements have not yet been performed on such samples but photoluminescence measurements at 4 K for the same SL grown by MBE show a maximum value around 1.42 eV, in good agreement with the simulated absorption thresholds (Figure 5).

## Conclusions

In conclusion, we have studied the optical properties of ultra thin II-VI quantum well structures suited for solar application and shown that a strong and stable optical process can occur at wavelengths of 885 nm. Further engineering of the electronic structure could be achieved by considering the different well thicknesses and alloyed materials in the superlattices. Our results show the usefulness of II-VI semiconductors to implement type-II band alignment in photovoltaic-based systems.

## Abbreviations

E: conduction (Electron) band state; HH: Heavy-Hole valence band state; LH: Light-Hole valence band state; MBE: Molecular Beam Epitaxy; SL: Superlattice; TB: Tight-Binding; VBO: Valence Band Offset.

## Competing interests

The authors declare that they have no competing interests.

## Authors’ contributions

SB carried out the absorption simulation of II-VI SL, CR introduced absorption calculation in the TB program, and JMJ wrote the TB simulation tool. JE and JMJ managed the simulation team. LG and RA performed the MBE growth and JB and JPR performed the optical measurements. RA, HM, and JB authored a patent which proposes type-II SL for solar cell [11]. All authors read and approved the final manuscript.
